# Olfactory bulb 6-OHDA neurotoxicity as a model of early cortical dysfunction

**DOI:** 10.1007/s11010-026-05591-1

**Published:** 2026-06-16

**Authors:** Edson Fiorenza-Neto, Bruna B. Gattiboni, Karin Almeida, Luis F. Marqueze, Bassam F. Mogharbel, Juliana R. N. de Aguiar, Larissa C. Marques, Larissa R. Lazarini, Stephanie R. S. Carvalhal, Angelica Boldt, Ricardo C. Cunha, Katherine A. T. Carvalho, Anand Thirupathi, Zsolt Radak, Ricardo A. Pinho

**Affiliations:** 1 Graduate Program in Health Sciences, School of Medicine and Life Sciences, Pontifical Catholic University of Parana, Curitiba, Brazil; 2 Pelé Pequeno Príncipe Research Institute, Child and Adolescent Health Research & Pequeno Príncipe Faculties, Advanced Therapy and Cellular Biotechnology in Regenerative Medicine Department, Curitiba, Brazil; 3 Department of Genetics, Federal University of Parana (UFPR), Curitiba, Brazil; 4 Faculty of Sports Science, Ningbo University, Ningbo, China; 5 Universidade Positivo, Curitiba, Brazil; 6 Research Institute of Sport Science, Hungarian University of Sports Science, Budapest, Hungary; 7 Laboratory of Exercise Biochemistry in Health (BioEx), Graduate Program in Health Sciences, School of Medicine and Life Sciences, Pontifícia Universidade Católica do Paraná, Block 11, Laboratory 2. Imaculada Conceição Street, 1155, Prado Velho, Curitiba, PR 80215-901 Brazil

**Keywords:** Olfactory bulb, 6-Hydroxydopamine, Parkinson’s disease, Inflammation, Oxidative stress

## Abstract

The olfactory bulb (OB) is one of the earliest brain regions affected in neurodegenerative diseases such as Parkinson’s disease (PD). Its high metabolic rate, dopaminergic modulation, and connectivity with the cortical and limbic regions make it particularly vulnerable to early neuroinflammatory and oxidative processes. This study aimed to investigate whether the administration of 6-hydroxydopamine (6-OHDA) into the OB induces behavioral, redox, and inflammatory alterations associated with early cortical disturbances. Male Wistar rats were randomly assigned to the sham or 6-OHDA groups and underwent stereotaxic injection of the vehicle or 6-OHDA into the left OB. Behavioral performance was assessed in the open-field test 12 days after surgery, and cortical tissue was collected for biochemical and molecular analyses. Cytokines (IL-1β, IL-6, TNF-α, IL-10, IFN-γ, and MCP-1) were quantified by Luminex, redox parameters (CAT, GPx, MDA, and protein carbonyls) by spectrophotometry, and neuronal signaling proteins (c-FOS, CREB, and BDNF) by qPCR. Animals with 6-OHDA lesions exhibited decreased latency and increased time spent in the central zone of the open field, indicating altered exploratory behavior. IL-6 levels were significantly elevated, whereas IFN-γ was reduced in the cortex, while IL-1β, TNF-α, MCP-1, and IL-10 remained unchanged. The oxidative stress markers MDA and protein carbonyls were increased, while catalase and glutathione peroxidase activities showed no change. The expression of c-FOS, CREB, and BDNF was not significantly modified. These findings indicate that localized 6-OHDA administration in the OB is sufficient to elicit behavioral, inflammatory, and oxidative alterations in connected cortical regions, which may resemble early non-motor features associated with olfactory dysfunction, without representing a neurodegenerative disease-specific process.

## Introduction

The olfactory bulb (OB) is a plastic structure that plays a role in sensory processing, neural integration, and connectivity with limbic and cortical regions. These OB functions influence cognition, emotion, and memory [1, 2]. Because of the continuous neurogenesis, high metabolic demand, and extensive synaptic remodeling, the OB is particularly susceptible to pathological insults. Increasing evidence indicates that OB dysfunction is among the earliest manifestations of several neurodegenerative diseases. In Parkinson’s disease (PD), olfactory impairment precedes motor symptoms by years and is considered one of the most consistent prodromal markers [3, 4]. Similar deficits in olfactory processing have also been reported in Alzheimer’s disease [5] and Huntington’s disease [6], reinforcing the idea that OB-related networks are particularly vulnerable to neurodegenerative processes and may serve as an entry point for the propagation of pathology to higher-order cortical regions [1].

Experimental models have been increasingly employed to investigate the vulnerability of OB and its contribution to the early stages of neurodegenerative diseases. Among these, the administration of 6-hydroxydopamine (6-OHDA), a selective catecholaminergic neurotoxin that has been applied to the nigrostriatal system to reproduce motor deficits characteristic of PD [7, 8], when administered in the intrabulbar region, causes partial dopaminergic depletion in periglomerular neurons, leading to impaired olfactory discrimination and disturbances in OB [9] and local neuroinflammation and partial loss of neurons expressing tyrosine hydroxylase (TH^+^) [10]. 6-OHDA also triggers non-motor manifestations such as depressive-like behaviors alongside olfactory deficits [11]. Previous studies have reported that 6-OHDA-induced lesions in the OB are characterized by redox imbalance and alterations in neurotransmission that progressively affect the cortical circuits [9, 10]. These findings support the use of OB-targeted 6-OHDA lesions to explore early functional and molecular consequences of localized catecholaminergic disruption. In this context, this study aimed to investigate whether administration of 6-OHDA into the OB induces behavioral, redox, and inflammatory alterations associated with early cortical disturbances. Thus, this study focused on olfactory-associated cortical regions to investigate how 6-OHDA administration in the OB might trigger molecular and functional alterations, thereby enabling the assessment of potential downstream effects across cortical regions.

## Materials and methods

### Bioethical information and experimental design

All experiments were conducted in accordance with the Brazilian College of Animal Experimentation and ARRIVE 2.0 standards and ethical principles. The data presented in this study were derived from a larger project approved by the Ethics Committee for the Use of Animals of the Federal University of Paraná (UFPR) under protocol 23075.072737/2022-70. Male Wistar rats (90 days old, 300–350 g) were obtained from the UFPR animal facility (Curitiba, Brazil), and they were immediately housed in polypropylene cages (≤ 4 per cage) under controlled conditions (25 °C, 12-h light/dark cycle) with ad libitum access to food and water. Rats were assigned to two experimental groups (Sham and 6-OHDA) and underwent stereotaxic surgery for vehicle or 6-OHDA injection into the OB. Behavioral assessments were performed 12 days after surgery [12]. The animals were euthanized on day 15, and cortical tissue was collected for subsequent analyses of inflammatory mediators, neuronal signaling markers, and redox parameters.

### Inclusion/exclusion criteria and final experimental group composition

A total of 30 animals were initially included in the study. No randomization was performed to allocate subjects to the experimental groups. Pre-established exclusion criteria included: death during surgery or immediately after the stereotaxic procedure, the development of severe clinical complications during the experimental period, such as marked weight loss (> 20%), persistent infection, or impaired recovery. Five animals died during or immediately after surgery and were excluded before behavioral assessments. Additionally, two animals were excluded during the experimental period due to clinical complications. Excluded animals were not replaced. Therefore, the final group composition consisted of the animals that survived surgery and met the predefined inclusion criteria. Sample sizes (10 animals per group for behavioral assessments, 6 animals per group for inflammatory and oxidative stress analyses, and 5 animals per group for gene expression analysis) were determined based on our previous experience with the nigrostriatal 6-OHDA model in our laboratory [13, 14], which has shown comparable variability patterns. Post-hoc power estimation (α = 0.05, power = 0.80) indicated sensitivity to detect large effects (Cohen’s d ~ 1.0), consistent with the variability typically observed in 6-OHDA models.

### 6-OHDA model

The neurotoxin 6-OHDA is taken up not only by the dopamine transporter but also by the norepinephrine transporter, which may result in unintended noradrenergic degeneration [15]. Therefore, animals were pretreated with imipramine hydrochloride (25 mg/kg, i.p.) 30 min before anesthesia induction to reduce noradrenergic uptake of 6-OHDA [16]. However, because it has lower selectivity than desipramine, incomplete noradrenergic protection cannot be ruled out [17]. Under ketamine (90 mg/kg) and xylazine (10 mg/kg) anesthesia (i.p.), the 6-OHDA (6 µg/µL; Sigma-Aldrich, St. Louis, MO, USA; H4381) was stereotaxically injected (2 µL of 0.02% ascorbic acid solution) into the left OB at the following coordinates: AP + 7.5 mm, ML ± 1.5 mm, DV − 3.0 mm, relative to the bregma and the dural surface. The sham group received an injection of 0.02% ascorbic acid in saline and served as the control. The injection rate for both 6-OHDA and vehicle was 0.2 µL/min, and the syringe was left in place for an additional 2 min after injection to ensure complete diffusion to the target site. The needle was then slowly withdrawn, the incision was sutured, and the animals were returned to their cages. Postoperative care was provided for 5 days following surgery using dipyrone at a dose of 100 mg/kg/day.

### Olfactory discrimination test (ODT)

Olfactory discrimination was evaluated on days 12–13 after stereotaxic surgery using a familiar versus non-familiar odor preference task, adapted from Schellinck et al. [18]. The test was conducted in the same open-field arena (50 × 50 × 40 cm), which was lined with clean paper. For each animal, bedding was collected from its own home cage and placed in one half of the arena (familiar odor), while the opposite half contained clean, unused bedding (non-familiar odor). Each rat (*n* = 10 per group) was positioned in the center of the arena and allowed to freely explore both compartments for 5 min under controlled temperature (22 ± 2 °C) and low illumination. The time spent in each compartment was recorded by an observer who was blinded to the group allocation. The preference for the non-familiar odor was interpreted as a preserved olfactory discrimination.

### Open field maze (OFM) test

The OFM was conducted on days 14–15 after 6-OHDA administration to evaluate exploratory and locomotor behavior. Each animal was placed individually in the center of a square arena (50 × 50 cm, 40 cm high, with an opaque floor) under controlled illumination for 5 min. The arena was divided into equal quadrants (5 × 5 cm), with the four central quadrants defined as the central zone and the remaining outer quadrants as the peripheral zone. Latency to leave the central zone, the number of rearings, and the time spent in the central and peripheral zones were recorded by direct observation from a single researcher. The procedure followed the methodological recommendations for the OFM [19].

### Euthanasia and tissue collection

Animals were euthanized on day 15 after 6-OHDA administration for tissue collection. The animals were anesthetized with ketamine (80 mg/kg) and xylazine (10 mg/kg, i.p.) and euthanized by decapitation using a guillotine. Cortical tissue associated with olfactory processing was collected from the anterior portion of the brain, extending from the frontal pole to approximately AP − 2.45 mm (relative to bregma) [20], including regions such as the piriform cortex and nearby frontal cortical areas. No microdissection of specific subregions was performed, and subcortical structures were not included. The samples were rinsed in ice-cold saline solution, blotted dry on Whatman filter paper, and immediately transferred to additive-free microtubes. The samples were immersed in liquid nitrogen and subsequently stored at − 80 °C until biochemical analyses were performed. The remaining tissues and carcasses were placed in opaque white plastic bags and kept frozen until collection and final disposal at a licensed sanitary landfill, in accordance with the Brazilian Sanitary Surveillance regulations.

### Cytokine and chemokine analysis by Luminex

Approximately 20 mg of cortical tissue was homogenized in radioimmunoprecipitation assay buffer (RIPA) buffer containing protease and phosphatase inhibitors and centrifuged at 12,000 rpm for 15 min at 4 °C. Protein concentration was determined in the supernatant using the Bradford assay. Cytokine and chemokine levels were quantified using a multiplex magnetic bead assay (MILLIPLEX^®^ Rat Expanded Cytokine/Chemokine Magnetic Bead Panel, Millipore Sigma), according to the manufacturer’s instructions. The panel included pro-inflammatory mediators (tumor necrosis factor TNF-α, interleukins (IL)-1β and IL-6, monocyte chemoattractant protein (MCP)-1, and interferon (IFN)-γ and the anti-inflammatory cytokine IL-10. The selected markers were chosen based on their well-established involvement in neuroinflammatory processes associated with dopaminergic and olfactory system dysfunction, particularly in experimental models of 6-OHDA-induced neurodegeneration [7, 21, 22]. Plates were read on a Luminex detection system (MAGPIX, Life Technologies, Carlsbad, CA, USA), and cytokine concentrations were calculated from standard curves using the manufacturer’s software. Data were normalized to total protein content and expressed as pg/mg protein.

### Redox parameters by spectrophotometry

Approximately 20–30 mg of cortical tissue were homogenized (1:10, w/v) in phosphate buffer (50 mM, pH 7.0) using a Potter–Elvehjem homogenizer. Homogenates were centrifuged at 12,000×*g* for 5 min at 4 °C, and the resulting supernatants were used for spectrophotometric determinations. Protein concentration was measured by the Bradford method for normalization. Catalase (CAT) and glutathione peroxidase (GPx) activities were determined according to Weydert and Cullen [23], by monitoring the decomposition rate of hydrogen peroxide at 240 nm and the oxidation of nicotinamide adenine dinucleotide phosphate (NADPH) at 340 nm, respectively. Protein carbonyl content was measured as an index of protein oxidation as described by Colombo et al. [24], based on the reaction with 2,4-dinitrophenylhydrazine (DNPH) and detection at 370 nm. Lipid peroxidation was evaluated by quantifying malondialdehyde (MDA) levels using a commercial TBARS (Thiobarbituric Acid Reactive Substances) Assay Kit, Sigma-Aldrich (MAK085, St. Louis, MO, USA), according to the manufacturer’s instructions. All results were normalized to total protein content and expressed as enzyme activity units or concentration in mmol per mg of protein.

### Gene expression analysis by RT-qPCR

Total RNA was extracted from cerebral cortex samples using the Quick-RNA™ Miniprep Kit (Zymo Research). RNA purity and concentration were assessed by spectrophotometry (NanoDrop One, Thermo Fisher Scientific, Waltham, MA, USA) and fluorimetry (Qubit Flex, Thermo Fisher Scientific, Waltham, MA, USA), and integrity was verified by absorbance ratios at 260/280 and 260/230 nm. Complementary DNA (cDNA) was synthesized from total RNA using the High-Capacity cDNA Reverse Transcription Kit (Thermo Fisher Scientific). Primer efficiency and specificity were previously validated in independent assays using serial dilutions of pooled complementary DNA (cDNA). The expression of target genes (*Creb -* cAMP responsive element binding protein 1, *Fos -* FBJ osteosarcoma oncogene, and *Bdnf* - brain-derived neurotrophic factor) and endogenous controls (*Hprt1* - hypoxanthine phosphoribosyltransferase 1) was quantified by real-time quantitative PCR (RT-qPCR) using both 384-well and 96-well plates, with *Actb* (beta-actin) as the reference gene (Table 1). Reactions were performed with 12.5 ng/µL or 50 ng/µL cDNA, depending on the assay. Relative gene expression was calculated using the 2(-Delta Delta C(T)) method, with the Sham group as the calibrator.

**Table 1:** Primer sequences used for RT-qPCR analysis

Gene	Forward primer (5′→3′)	Reverse primer (5′→3′)	Reference/source
*Hprt1*	CTCATGGACTGATTATGGACAGGA	GCAGGTCAGCAAAGAACTTATAGCC	Endogenous
*Actb*	AGCCATGTACGTAGCCATCCA	TCTCCGGAGTCCATCACAATG	Endogenous
*Creb1*	GTTGTTATGGCGTCCTC	ATTCTCTTGCTGCTTCC	Target gene
*Fos*	ACGGAGAATCCGAAGGGAAAGGAA	TCTGCAACGCAGACTTCTCGTCTT	Target gene
*Bdnf*	CAAGGCAACTTGGCCTACCC	GAGCATCACCCGGGAAGTGT	Target gene

All primers were designed from rat mRNA sequences (GenBank, NCBI) and validated for specificity and efficiency prior to amplification

*Hprt1* hypoxanthine phosphoribosyltransferase 1, *Actb* beta-actin, *Creb1* cAMP responsive element binding protein 1, *Fos* FBJ osteosarcoma oncogene, *Bdnf* brain-derived neurotrophic factor

### Statistical analysis

Data were tested for normality using the Shapiro–Wilk test. For comparisons between two independent groups, parametric data were analyzed using a two-tailed unpaired Student’s t-test, whereas non-parametric data were analyzed using the Mann–Whitney *U* test. For the olfactory discrimination test, data were analyzed using a two-way ANOVA (group × odor type), followed by Tukey’s post hoc test. Results are presented as mean ± standard deviation (SD) for parametric data or as median and interquartile range (IQR) for non-parametric data. Statistical analyses were performed using GraphPad Prism version 10.0 (GraphPad Software, San Diego, CA, USA). A p-value ≤ 0.05 was considered statistically significant.

### Artificial intelligence use

ChatGPT (version 5.0, OpenAI) and Grammarly were used to assist with manuscript organization and language refinement (basic checks of grammar, spelling, and punctuation), while Zotero was used to format citations and references. The AI-generated outputs were critically reviewed, verified, and edited by the authors to ensure scientific accuracy and consistency with the study’s data and interpretations. All scientific content, including methodological descriptions, data analyses, results, discussion, and conclusions, represents the authors’ original work and intellectual contribution.

## Results

### Animal behavioral analysis

**Fig. 1:**
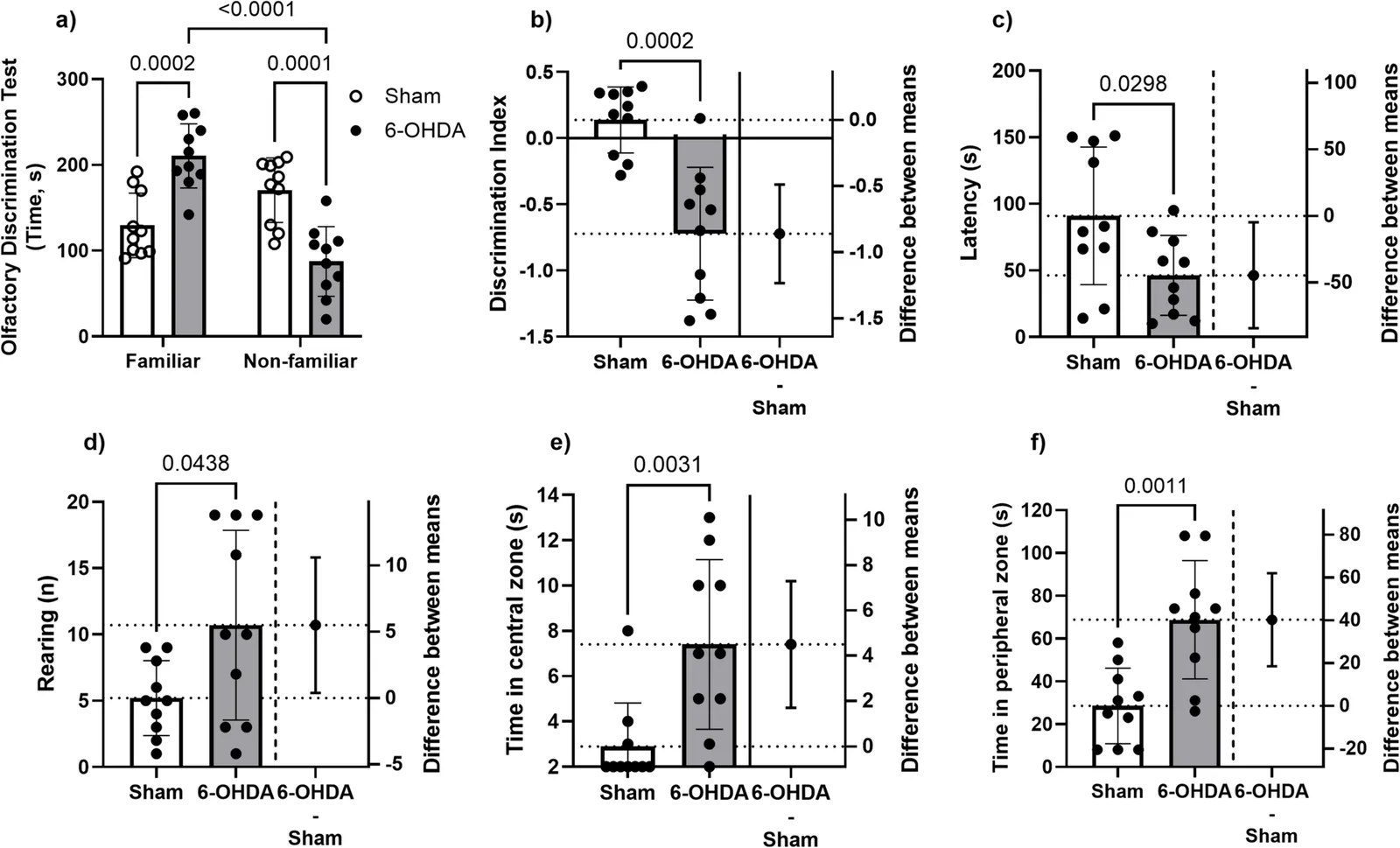
Behavioral analysis showing **a** olfactory discrimination test, **b** olfactory discrimination index, **c** latency to enter the central zone, **d** number of rearing, **e** time spent in the central zone, and **f** time spent in the peripheral zone in Sham and 6-OHDA groups (*n* = 10 per group). Data are expressed as mean ± SD, with individual values represented by dots. Statistical comparisons were performed using a two-way ANOVA followed by Tukey’s post hoc test (**a**), the unpaired Student’s *t*-test for panels (**b**, **d**–**f**), and the Mann–Whitney U test for panel (**c**). *P-values* indicate significant differences between groups. 6-OHDA − 6-hydroxydopamine

Behavioral assessments were conducted 12 days after the stereotaxic surgery (Fig. 1). The olfactory discrimination test (ODT) revealed significant deficits in odor recognition in 6-OHDA–treated rats, as evidenced by reduced preference for the non-familiar odor and a lower discrimination index compared to the Sham group. Two-way ANOVA revealed a significant group × odor interaction (F(1,36) = 45.92, *p* < 0.0001). Tukey’s post hoc test showed that 6-OHDA animals differed from Sham rats in both the familiar (*p* = 0.0002) and non-familiar conditions (*p* = 0.0001). Moreover, 6-OHDA animals exhibited a significant difference between familiar and non-familiar odors (*p* < 0.0001), whereas no significant difference was observed within the Sham group (*p* = 0.0960). Consistently, the discrimination index was markedly reduced in 6-OHDA rats (Fig. 1b; mean difference = − 0.86, 95% CI − 1.23 to − 0.49; *p* = 0.0002), indicating impaired olfactory discrimination. In the open-field test, the latency to enter the central zone (Fig. 1c) was significantly reduced in 6-OHDA-lesioned rats (mean difference = − 44.6, 95% CI − 84.3 to − 4.9; *p* = 0.0298), with a mean difference of − 44.6 s. In contrast, the number of rearings (Fig. 1d) was higher in the 6-OHDA group (mean difference = 5.5, 95% CI 0.18 to 10.8; *p* = 0.0438). Moreover, 6-OHDA animals spent more time both in the central zone (Fig. 1e; U = 13; *p* = 0.0031) and in the peripheral zone (Fig. 1f; mean difference = 40.3, 95% CI 18.5 to 62.1; *p* = 0.0011) compared with Sham rats.

### Inflammatory analysis

**Fig. 2:**
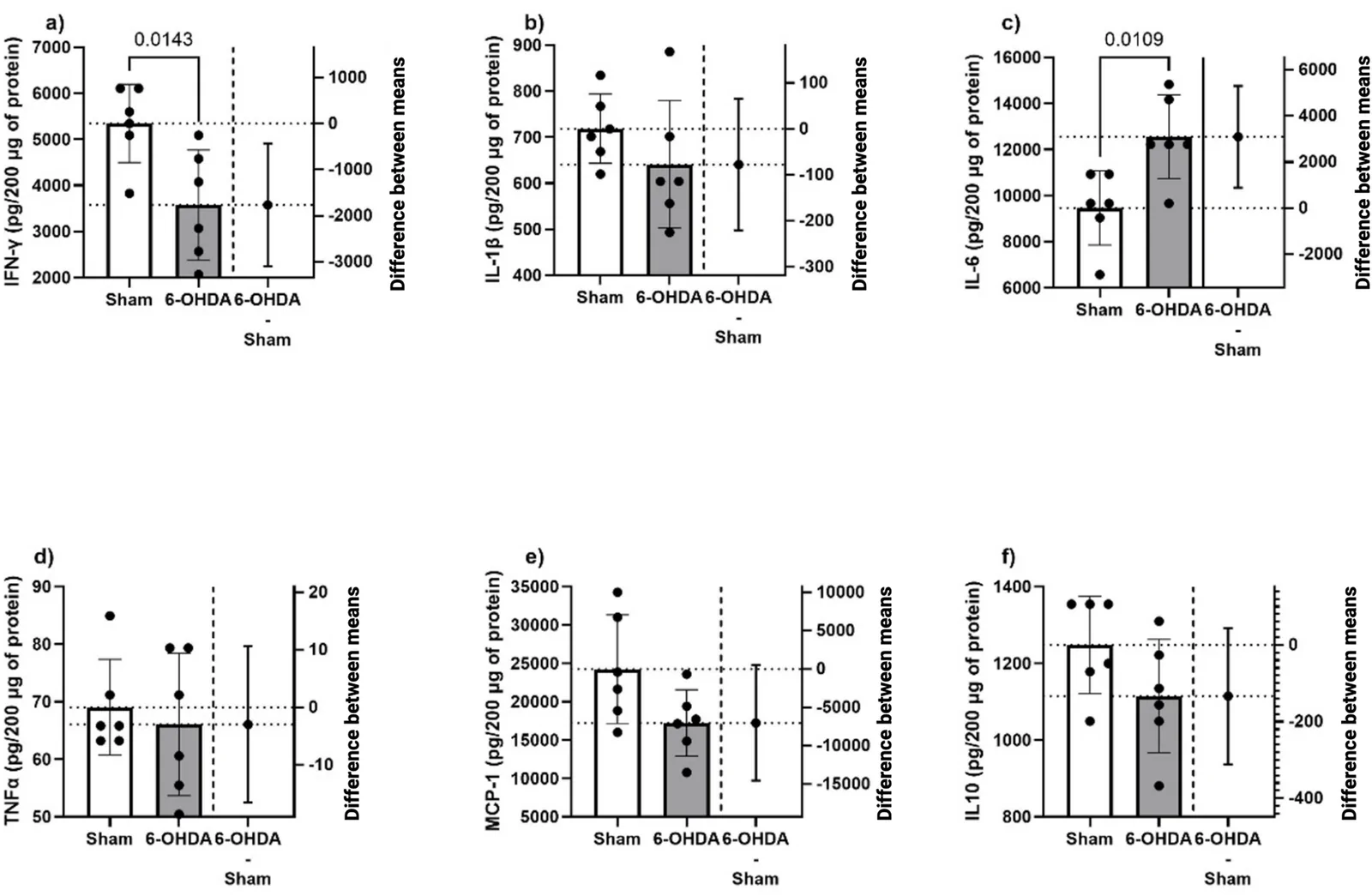
Cytokine levels in the cortical tissue after 6-OHDA administration. Protein concentrations of **a** IFN-γ, **b** IL-1β, **c** IL-6, **d** TNF-α, **e** MCP-1, and **f** IL-10 in Sham and 6-OHDA groups (*n* = 6 per group). Data are expressed as mean ± SD, with individual values represented by dots. Statistical comparisons were performed using the unpaired Student’s T-test. *P-values* indicate significant differences between groups. *6-OHDA* 6-hydroxydopamine, *INF* interferon, *IL* interleukin, *TNF* tumor necrosis factor, *MCP* monocyte chemoattractant protein

Inflammatory mediators were assessed as shown in Fig. 2. The concentration of IFN-γ (Fig. 2a) was significantly lower in the 6-OHDA group compared with the Sham group (mean difference = − 1768, 95% CI − 3098 to − 437.8; *p* = 0.0143). In contrast, IL-6 levels (Fig. 2c) were significantly higher in the 6-OHDA group (mean difference = 3088, 95% CI 880.0 to 5295; *p* = 0.0109). No statistically significant differences were observed between groups for IL-1β (Fig. 2b), TNF-α (Fig. 2d), MCP-1 (Fig. 2e), or IL-10 (Fig. 2f).

### Neural signaling

**Fig. 3:**
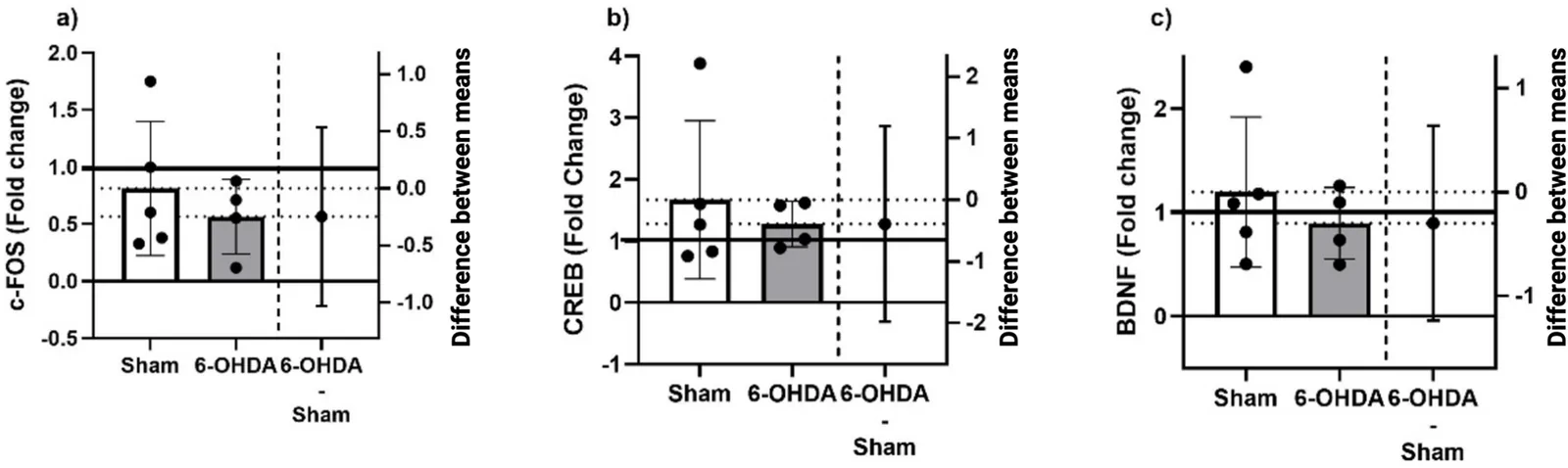
Expression of neuronal signaling-related proteins in the cortical tissue after 6-OHDA administration. Gene expression of **a** *Fos*, **b** *Creb*, and **c** *Bdnf* in Sham and 6-OHDA groups (*n* = 5 per group). Data are expressed as fold change relative to the mean value of the Sham group and presented as mean ± SD, with individual values represented by dots. Statistical comparisons were performed using the unpaired Student’s *t*-test. *6-OHDA* 6-hydroxydopamine; *Creb1* cAMP responsive element binding protein 1; *Fos* FBJ osteosarcoma oncogene; *Bdnf* brain-derived neurotrophic factor

The expression of genes related to neuronal signaling in the cortex was measured, as shown in Fig. 3. No significant differences were observed between groups for *Fos* (Fig. 3a), *Creb* (Fig. 3b), or *Bdnf* (Fig. 3c) expression levels. Data are presented as fold change relative to the Sham group.

### Oxidative stress markers

**Fig. 4:**
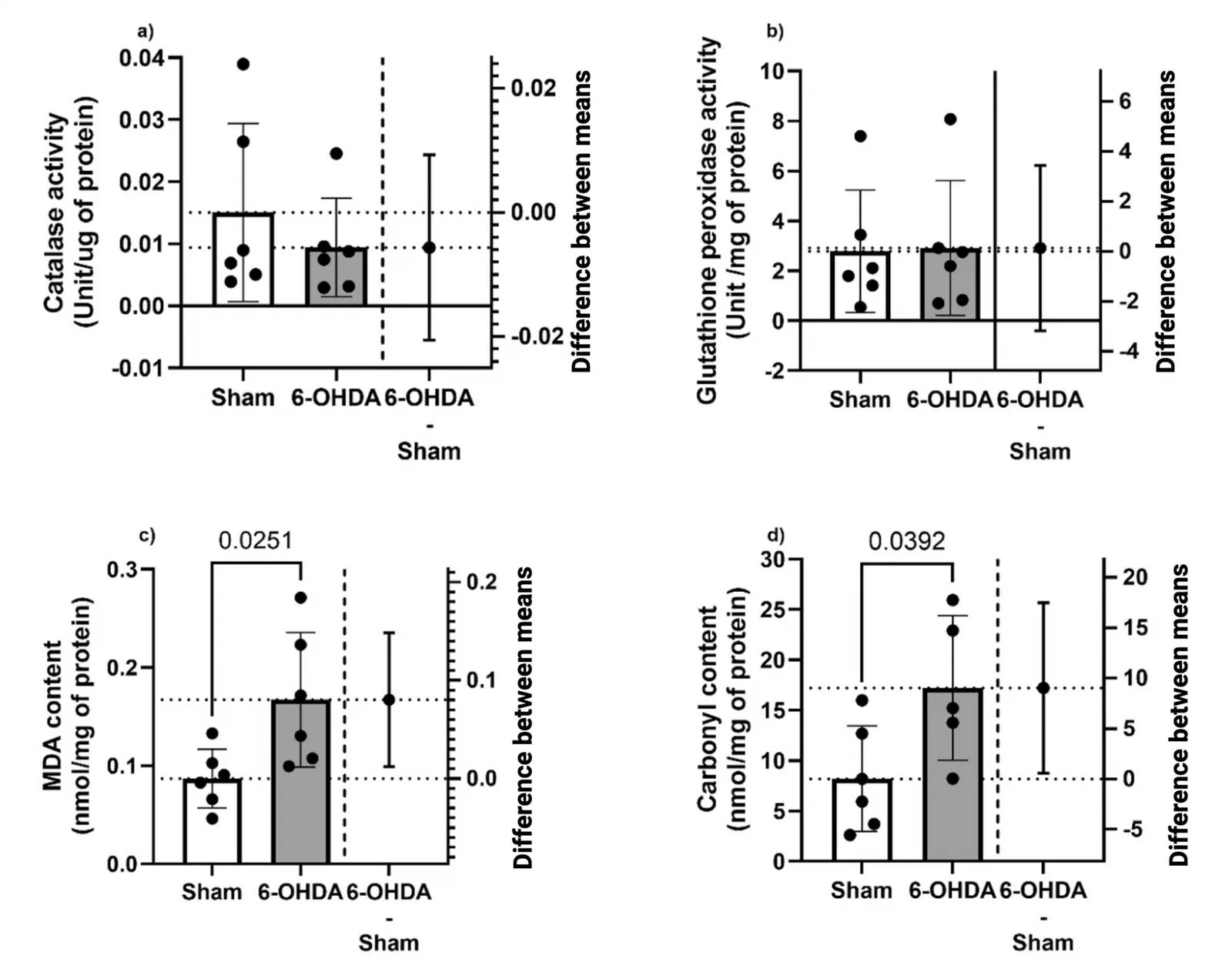
Redox parameters in the cortical tissue after 6-OHDA administration. Biochemical analysis of **a** catalase activity, **b** glutathione peroxidase (GPx) activity, **c** malondialdehyde (MDA) content, and **d** carbonyl protein content in Sham and 6-OHDA groups (*n* = 6 per group). Data are expressed as mean ± SD, with individual values represented by dots. Statistical comparisons were performed using the unpaired Student’s *T*-test. *P* values indicate significant differences between groups. *6-OHDA* 6-hydroxydopamine

The results of redox parameters are presented in Fig. 4. No significant differences were detected between groups for catalase (Fig. 4a) or glutathione peroxidase (GPx) activities (Fig. 4b). In contrast, MDA (Fig. 4c, mean difference = 0.08, 95% CI 0.012 to 0.015; *p* = 0.0251) and protein carbonyl (Fig. 4d, mean difference = 9.01, 95% CI 0.56 to 17.47; *p* = 0.0392) levels were significantly higher in the 6-OHDA group compared with the Sham group.

## Discussion

In the present study, administration of 6-OHDA led to a clear deficit in odor discrimination. Lesioned animals showed reduced preference for non-familiar bedding and a decreased discrimination index, indicating early impairment of olfactory processing. These findings are consistent with previous studies reporting that 6-OHDA-induced lesions in the OB can lead to olfactory deficits in rodents [11, 25]. These data suggest that 6-OHDA may affect OB function, as inferred from the observed behavioral alterations, although this was not directly measured in the present study and does not provide evidence of dopaminergic neuron loss or lesion specificity at the OB level. Therefore, the inflammatory and oxidative changes observed here should be interpreted as downstream responses in cortical regions functionally connected to the olfactory bulb, rather than as direct effects within the OB itself.

6-OHDA–lesioned rats also showed a reduced latency to enter the central zone and increased time spent in the center of the open field, indicating modifications in exploratory behavior. Lesions restricted to the OB have previously been associated with early behavioral and emotional alterations in models of PD [9, 11]. In this context, the present results suggest that subtle changes in locomotor and exploratory patterns following 6-OHDA administration in the OB are associated with changes consistent with catecholaminergic disruption, which can precede the appearance of motor deficits typically observed in nigrostriatal models, as reported in previous studies by our group and others [8, 26].

Reduced latency and enhanced central exploration may reflect alterations in anxiety-related behavior, as previously reported following experimental manipulation of olfactory structures [10, 11]. In addition, increased locomotor activity and rearing behavior suggest a broader change in exploratory activity rather than an isolated anxiety-like response observed in the open field. Rearing is commonly interpreted as a measure of vertical exploration and environmental information-gathering behavior, particularly in response to novelty, although its interpretation may vary across experimental contexts [19, 27, 28]. Thus, the observed behavioral profile is consistent with enhanced exploratory activity and environmental interaction following dopaminergic disruption, although a contribution of altered emotional reactivity cannot be excluded. Although both anxiety and motivation can influence open-field parameters, they are recognized as sensitive indicators of early neurochemical disturbances induced by dopaminergic injury [7, 8]. These behavioral modifications may result from disrupted monoaminergic signaling within interconnected cortical and limbic circuits, which are functionally connected to the OB [9]. Previous evidence has revealed that dopaminergic depletion in olfactory regions can lead to emotional and motivational alterations, even in the absence of overt motor symptoms [1, 11] preceding the classical motor manifestations observed in neurodegenerative disease, such as PD [3, 4].

The initiation and propagation of non-motor symptoms have been recognized to be influenced by neuroinflammatory processes [29, 30]. In the present study, 6-OHDA-lesioned animals showed reduced IFN-γ and increased IL-6 concentrations in the cortical region, whereas no significant differences were detected for IL-1β, TNF-α, MCP-1, or IL-10. These findings suggest an early and selective cytokine imbalance that may reflect non-specific cortical immune responses in functionally connected regions. Cytokine alterations within olfactory structures are consistent with previous reports indicating that inflammatory signaling contributes to dopaminergic vulnerability and synaptic remodeling in early stages of the disease [10]. Rey et al. [1] suggest that the OB represents a potential entry and amplification site for prion-like propagation of pathological α-synuclein, where microglial activation and local cytokine release facilitate neuronal dysfunction. In addition, elevated IL-6 levels, as observed in this study, have been reported as a marker of early neuroinflammatory activity, capable of modulating glial reactivity and reflecting responses that may resemble those observed in neurodegenerative conditions [31].

Inflammatory responses are known to influence neuronal signaling, synaptic transmission, and neurotrophic factor expression in neurodegenerative diseases [32–34]. However, in the present study, no significant differences were found in the expression of *Fos*,* Creb*, or *Bdnf* in the cortex after 6-OHDA administration in the OB. Both c-FOS and CREB are immediate-early proteins that reflect neuronal activation and transcriptional activity. At the same time, BDNF is a key neurotrophin involved in synaptic maintenance and the survival of dopaminergic neurons. The absence of changes in these markers may indicate that, despite the local increase of IL6, the extent of dopaminergic injury at this early point was insufficient to alter neurotrophic or transcriptional signaling pathways. Alternatively, alterations in *Fos*,* Creb*, or *Bdnf* expression levels may occur at later stages, once neuronal degeneration or compensatory mechanisms become more established [14, 35, 36].

Neurochemical alterations induced by 6-OHDA are associated with inflammatory signaling and oxidative stress. The increased levels of MDA and protein carbonyls observed indicate enhanced oxidative damage without a concomitant compensatory upregulation of major antioxidant enzymes. The increase in oxidative markers is consistent with the known mechanism of 6-OHDA toxicity, in which the compound undergoes auto-oxidation and redox cycling, generating reactive oxygen species (ROS) and quinones that promote lipid and protein oxidation [37, 38]. The higher IL-6 levels observed in the same experimental condition may also contribute to oxidative stress, as IL-6 signaling has been shown to enhance ROS production and mitochondrial dysfunction in the central nervous system, via activation of NADPH oxidase, and downstream JAK/STAT3 pathways, leading to redox imbalance and neuronal vulnerability [39, 40]. While markers such as IL-6 and MDA reflect a general inflammatory and oxidative response to injury, their detection in cortical areas distant from the site of 6-OHDA administration suggests an early trans-synaptic or systemic vulnerability, which may reflect downstream effects of localized neural disruption, without implying disease-specific mechanisms of progression.

Together, these findings suggest that localized 6-OHDA administration in the OB is associated with early behavioral and molecular alterations in connected cortical regions. However, while this model reproduces specific behavioral and early molecular dysfunctions, it does not fully capture the disease’s progressive nature. Although NET inhibition was used to minimize noradrenergic damage, the possibility of partial noradrenergic involvement cannot be completely excluded. This model therefore provides an experimental framework for investigating early cortical consequences of 6-OHDA in the OB for testing interventions targeting inflammation and oxidative stress in the preclinical stages of neurodegeneration.

### Limitations

Although the present findings provide evidence that localized 6-OHDA administration to the OB induces behavioral and molecular alterations, certain methodological limitations should be considered. First, the nature and specificity of the primary effect within the OB were not directly validated, as dopaminergic depletion was inferred from the established effects of 6-OHDA but not confirmed by tyrosine hydroxylase immunohistochemistry or dopamine quantification. Accordingly, the effects observed should be interpreted as reflecting a localized catecholaminergic disruption rather than a selective dopaminergic lesion. In addition, the absence of direct anatomical or neurochemical validation limits the ability to determine the extent and effectiveness of the lesion at the primary site. Moreover, because manipulation was anatomically restricted to the OB, the cortical alterations observed here should be interpreted with caution, as they likely represent downstream responses in functionally connected regions rather than direct cortical involvement. Furthermore, cortical samples were obtained from relatively broad anterior regions without microdissection of specific subareas, which may have diluted region-specific molecular signals. Another limitation is the use of imipramine rather than desipramine as a norepinephrine transporter inhibitor, which, due to its lower selectivity, may have resulted in incomplete noradrenergic protection and contributed to a broader catecholaminergic effect. Finally, the relatively small sample size, particularly for molecular endpoints, may limit statistical power and generalizability. In addition, only male animals were used to reduce variability associated with the estrous cycle; however, this choice limits the generalizability of the findings and does not account for potential sex-specific differences.

## Data Availability

The data that support the findings of this study are available from the corresponding author upon reasonable request.
